# Efficient large fragment deletion in plants: double pairs of sgRNAs are better than dual sgRNAs

**DOI:** 10.1093/hr/uhad168

**Published:** 2023-08-22

**Authors:** Guoning Zhu, Lingling Zhang, Liqun Ma, Qing Liu, Kejian Wang, Jinyan Li, Guiqin Qu, Benzhong Zhu, Daqi Fu, Yunbo Luo, Hongliang Zhu

**Affiliations:** College of Food Science & Nutritional Engineering, China Agricultural University, Beijing, 100083, China; College of Food Science & Nutritional Engineering, China Agricultural University, Beijing, 100083, China; College of Food Science & Nutritional Engineering, China Agricultural University, Beijing, 100083, China; State Key Laboratory of Rice Biology and Breeding, China National Rice Research Institute, Chinese Academy of Agricultural Sciences, Hangzhou, 310006, China; State Key Laboratory of Rice Biology and Breeding, China National Rice Research Institute, Chinese Academy of Agricultural Sciences, Hangzhou, 310006, China; College of Food Science & Nutritional Engineering, China Agricultural University, Beijing, 100083, China; College of Food Science & Nutritional Engineering, China Agricultural University, Beijing, 100083, China; College of Food Science & Nutritional Engineering, China Agricultural University, Beijing, 100083, China; College of Food Science & Nutritional Engineering, China Agricultural University, Beijing, 100083, China; College of Food Science & Nutritional Engineering, China Agricultural University, Beijing, 100083, China; College of Food Science & Nutritional Engineering, China Agricultural University, Beijing, 100083, China

Dear Editor,

Clustered Regularly Interspaced Short Palindromic Repeats (CRISPR) is a powerful and versatile gene editing system that has been extensively utilized in various animals and plants, which holds enormous potential and value for scientific research and breeding. However, single-targeted CRISPR can only induce a few base deletions, insertions, or substitution. Ideally, these mutations result in premature termination of the protein encoded by the target gene, leading to a loss of function [1]. Nevertheless, in some cases, these mutations can produce truncated proteins whose still have function [2]. Moreover, when applied to long non-coding RNAs (lncRNAs), which rely on their secondary structures to function, single base mutations may not alter their structures so that they lose their function. Such imprecise genome editing models hinder the accurate identification of gene function. In crops with high gene editing efficiency, such as rice and soybean, dual sgRNAs can induce large fragment deletions on target genes, thereby avoiding these limitations [3, 4]. However, obtaining large fragment deletions through dual sgRNAs is inefficient or rarely reported in most horticultural crops, such as tomato [5]. Hence, efficient technologies for large DNA fragment deletions in horticultural crops are urgently needed for research and breeding purposes.

Recently, the use of dual sgRNAs near microhomologous sites (MHS) has been shown to enhance the efficiency of large fragment deletions in rice [6]. Microhomology-mediated end-joining (MMEJ) occurs between MHSs following DNA double-strand breaks [7]. To investigate the suitability of this approach in tomato, we designed dual sgRNAs near the MHSs on *SlyPDS*, *SlyRIN-MC*, and the intergenic regions of tomato chromosome 3 using the CRISPR-GE web-based tool (http://skl.scau.edu.cn/). After DNA extraction from tomato transgenic explants, PCR was performed using primers located outside the large DNA deletion region and sequenced. Unfortunately, only 5.1% (15/294) of the tomato explants had large DNA deletions (Fig. S1, see online supplementary material). Moreover, only 20% of the large deletions were mediated by MMEJ (Fig. S1A, see online supplementary material). Interestingly, the actual MHSs were shorter than the predicted MHSs in all of our MMEJ events (Fig. 1A). These results suggest that short MHSs are sufficient for MMEJ to occur in tomato, but the MMEJ frequency is significantly lower than that of NHEJ. Therefore, it is not feasible to obtain large fragment deletions in tomato by the MMEJ strategy.

**Figure 1:**
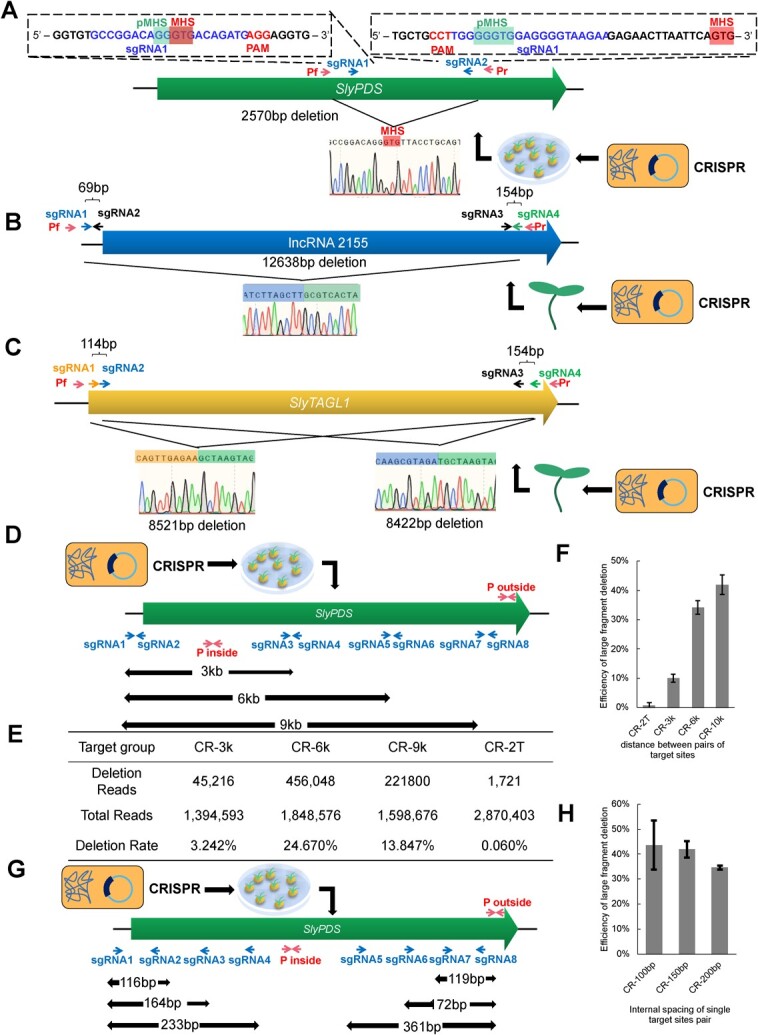
DPS improves deletion efficiency of large fragments in the tomato genome compared to dual sgRNAs. **A** Large fragment deletion of *SlyPDS* in the MMEJ strategy. The Pf/Pr primer pair detected large fragment deletions, and the predicted MHS are labeled as pMHS, while the actual repair sites are labeled as MHS. **B** Large fragment deletion of lncRNA2155 using DPS in transient transformation of tomato cotyledons. **C** Large fragment deletion of *slyTAGL1* using DPS in transient transformation of tomato cotyledons. **D** Target sites on *SlyPDS* were used to analyse the effect of the distance between pairs of target sites on the efficiency of large fragment deletion. Four target site groups were established: CR-2 T (sgRNA1, sgRNA8), CR-3 k (sgRNA1, sgRNA2, sgRNA3, sgRNA4), CR-6 k (sgRNA1, sgRNA2, sgRNA5, sgRNA6), and CR-9 k (sgRNA1, sgRNA2, sgRNA7, sgRNA8). The P outside and P inside primer pair were used for qPCR analysis to determine the efficiency of large fragment deletion. **E **Statistics of large fragment deletion reads in next generation sequencing. **F** qPCR analysis showed that DPS significantly improved the efficiency of large fragment deletion. The bars represent mean values ± SD. **G** Target sites on *SlyPDS* were used to analyse the effect of the internal spacing of single target site pairs on the efficiency of large fragment deletion. Three target site groups were established: CR-100 bp (sgRNA1, sgRNA2, sgRNA7, sgRNA8), CR-150 bp (sgRNA1, sgRNA3, sgRNA6, sgRNA8), and CR-200 bp (sgRNA1, sgRNA4, sgRNA5, sgRNA8). **H** qPCR analysis showed that the efficiency of large fragment deletion decreased with the increase in fragment length in a single target site pair. The bars represent mean values ± SD.

To enhance the efficiency of large fragment deletion, we have developed a new strategy that involves designing two pairs of target sites, referred to as double pairs of sgRNAs (DPS), at both ends of the target gene. Following this strategy, we designed two paired target sites at both ends of the tomato coding gene, *SlyTAGL1* [8], as well as for the long non-coding RNA lncRNA2155 [9] (Fig. 1B and C). After transient infection of tomato cotyledons, we analysed PCR products by electrophoresis and sequencing. We observed large deletions of the entire gene in 100% (7/7) and 87.5% (7/8) of the DNA samples of *SlyTAGL1* and lncRNA2155, respectively (Fig. S2A, see online supplementary material). Sequencing results indicated that for the large fragment deletion of lncRNA2155, only one type of deletion pattern between target site 1 and target site 4 was identified (Fig. 1B). However, for the large fragment deletion of *SlyTAGL1*, there were two types of events: the deletion between target site 1 and target site 4, and the deletion between target site 2 and target site 4 (Fig. 1C). Furthermore, we applied DPS to stable transformation of tomato explants and obtained a large number (37/72) of large fragment deletions on slyCh.03 (Fig. S2B, see online supplementary material). Our results demonstrate that DPS is an effective approach for obtaining large fragment deletions in plants.

To investigate more precisely the variables influencing large fragment deletion efficiency in order to improve it, we optimized the related parameters of the system, including the distance between pairs of target sites and the internal spacing of a single target site pair. To investigate the effect of the distance between target site pairs on the efficiency of large fragment deletion, we designed target site pairs with approximately 3 kb, 6 kb, and 9 kb spacing on the tomato *SlyPDS*, and also designed dual sgRNAs with 9 kb spacing at the same location (Fig. 1D). Both sgRNA1 and sgRNA8 were used in the dual sgRNA group as all deletions occurred between sgRNA1 and sgRNA8 in DPS with 9 kb spacing (Fig. S3, see online supplementary material). We took only the bud parts differentiated from the explants and removed the callus tissue, which belongs to the identification of stable transformation. After extracting the DNA from the buds, the concentration was measured and adjusted to the same level. Using primers designed within and across the deletion regions, large deletions were identified by targeted next-generation sequencing (NGS, BioProject ID: PRJNA1002368). Results showed that the deletion efficiency of DPS (3 kb, 6 kb, and 9 kb) was 3.242%, 24.670%, and 13.874%, respectively, while the deletion efficiency of dual target sites with 9 kb spacing was only 0.060% (Fig. 1E; Fig. S3, see online supplementary material).

Because the amplification efficiency of different primers may differ, we subsequently employed qPCR to reevaluate the efficiency of large fragment deletions. we first diluted the wild-type DNA template by 10-fold gradient before analysing the efficiency of primers. A standard curve for the number of primer cycles was made with different concentrations of template, from which the amplification efficiency of the primers was calculated. After that, the efficiency of large fragment deletion was calculated by the amplification efficiency of primer and Cq (Fig. S3, see online supplementary material).

The qPCR results showed that the deletion efficiency of dual target site pairs with different spacing (3 kb, 6 kb, and 9 kb) was 10.11%, 34.10%, and 41.99%, respectively, while the deletion efficiency of dual target sites with 9 kb spacing was only 0.69% (Fig. 1F). These results indicate that DPS can improve the efficiency of large fragment deletion by about 61-fold compared to dual sgRNAs. Moreover, the efficiency of large fragment deletion in DPS substantially increases with the increase of the distance between pairs of target sites, consistent with the dual sgRNA strategy [10].

To optimize the internal spacing of a single target site pair, target site pairs with internal spacing of about 100 bp, 150 bp, and 300 bp were designed on the *SlyPDS* (Fig. 1G). The qPCR results indicated that the deletion efficiency of different spacings (100 bp, 150 bp, 300 bp) was 43.67%, 41.99%, and 34.65%, respectively (Fig. 1H). Thus, widening the internal spacing of a single target site pair can slightly decrease the deletion efficiency.

Our results suggest that two key factors significantly influence the efficiency of large fragment deletions in DPS. Firstly, increasing the distance between pairs of target sites substantially enhances the efficiency of large fragment deletion. Secondly, increasing the internal spacing of single target sites pair can lead to a decrease in the efficiency of large fragment deletion. We also observed that target site pairs with opposite arrangements may produce longer deletions, though more data are needed to confirm this. Additionally, we generated stably transformed lncRNA1471 knockout plants utilizing DPS, wherein we identified 4.9% (5/103) of the T0 generation plants with large deletions, and homozygous plants with large deletions were obtained in the T1 generation. Furthermore, we implemented DPS in transient transformation of tobacco leaves and observed a marked improvement in deletion efficiency compared to the dual sgRNA strategy, which suggest that it has broader species applicability (Fig. S3, see online supplementary material). In conclusion, our study underscores the effectiveness of DPS as an efficient method for large fragment deletions in tomato, thereby enhancing the reliability of gene and lncRNA editing, facilitating functional genomics research, and genetic improvement.

## Supplementary Material

Web_Material_uhad168
