# Supplementary material for: Efficient large fragment deletion in plants: double pairs of sgRNAs are better than dual sgRNAs
Source: Hortic Res. 2023 Aug 22;10(10):uhad168. doi: 10.1093/hr/uhad168 (PMC10569238; doi:10.1093/hr/uhad168)
Supplement: Web_Material_uhad168 [file web_material_uhad168.zip › Figure S1.docx]

**A**

| Deletion site | Explants deletion rate% | Repair by MMEJ  rate% |
| --- | --- | --- |
| *SlPDS* | 1.9 (3/145) | 33.3 (1/3) |
| *SlRIN-MC* | 15.5 (11/71) | 0.9 (1/11) |
| Ch.03 | 1.4 (1/69) | 100 (1/1) |
| Total | 5.1 (15/294) | 20 (3/15) |

**B**


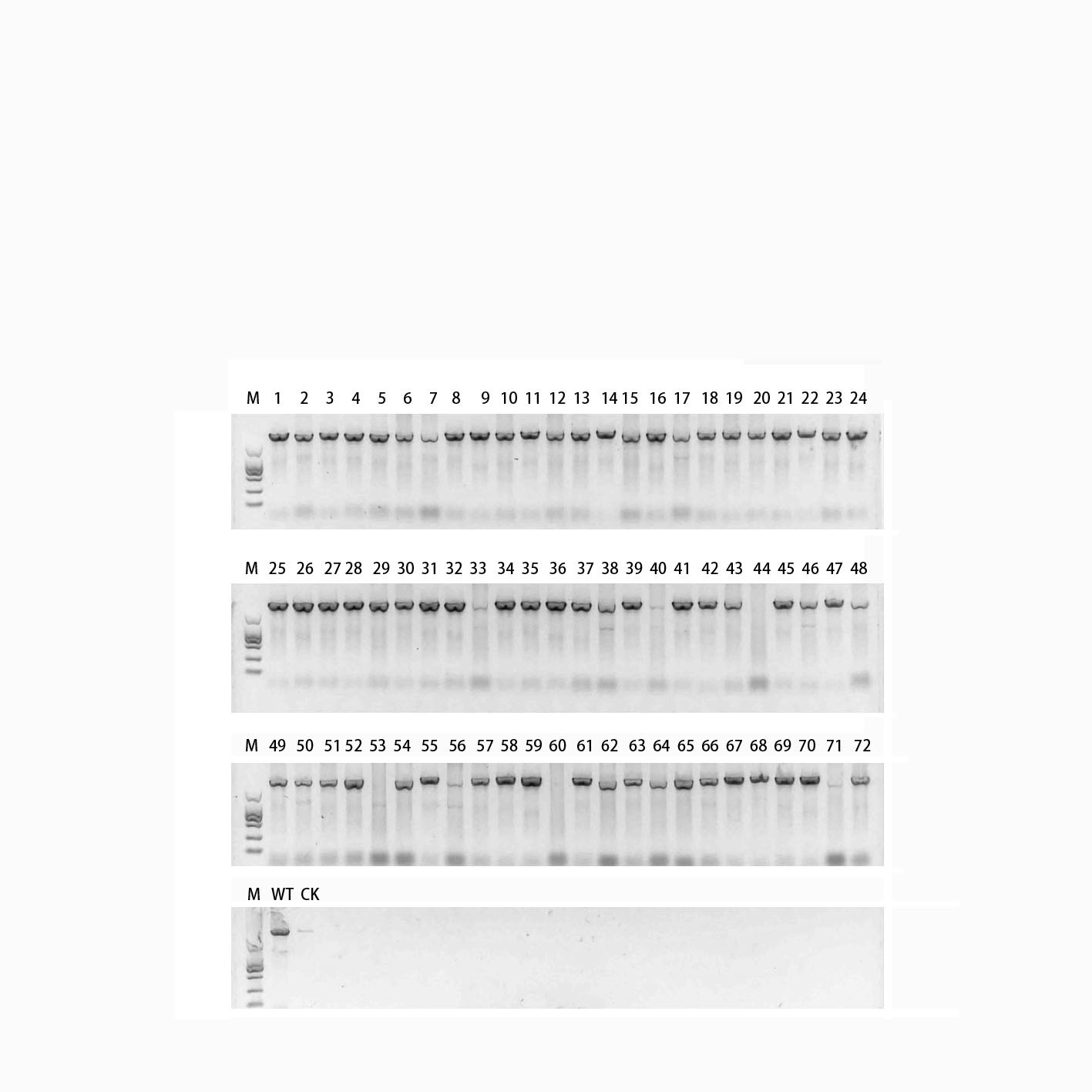


***slyPDS* (deletion)**

***slyPDS***

**C**

2000bp

1000bp

250bp

100bp

**
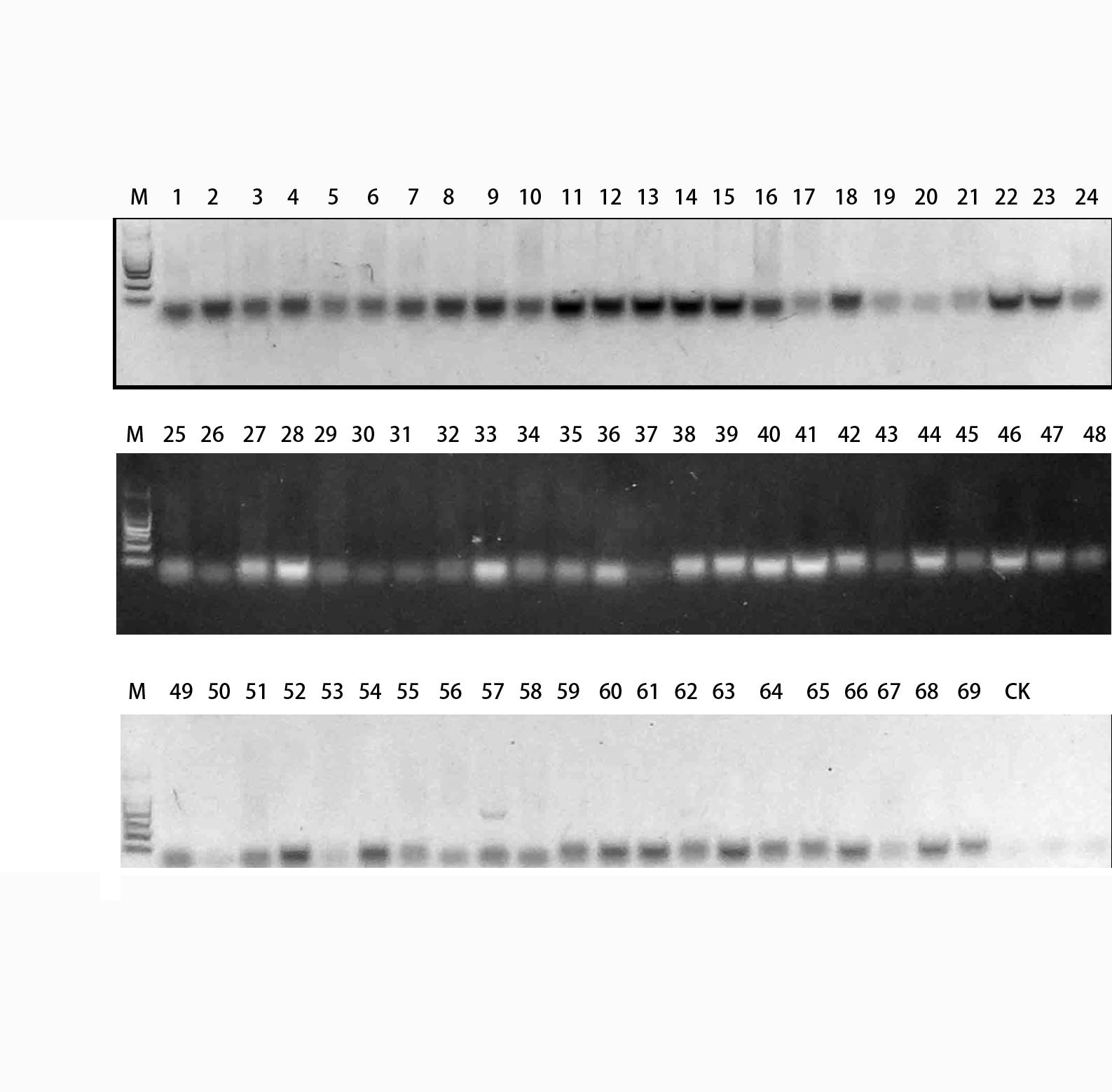
**

2000bp

1000bp

500bp

100bp

**SlyCh.03 (deletion)**

**D**

**
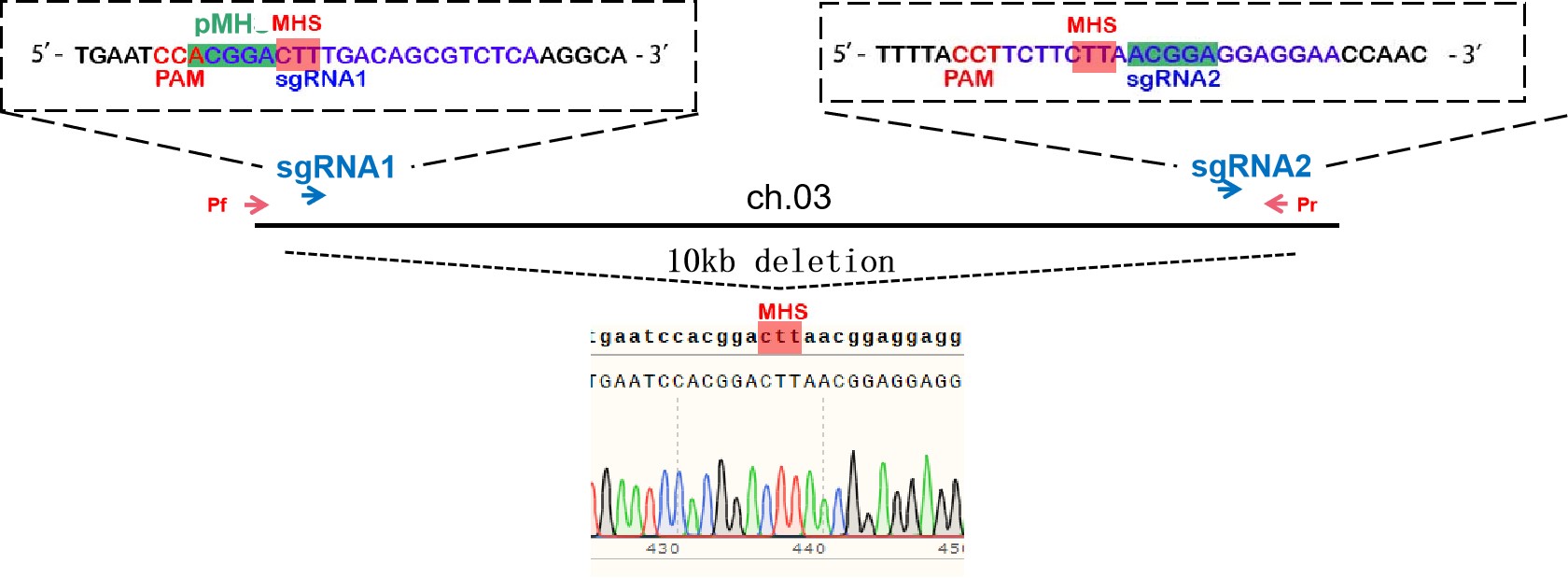
**

**E**

**
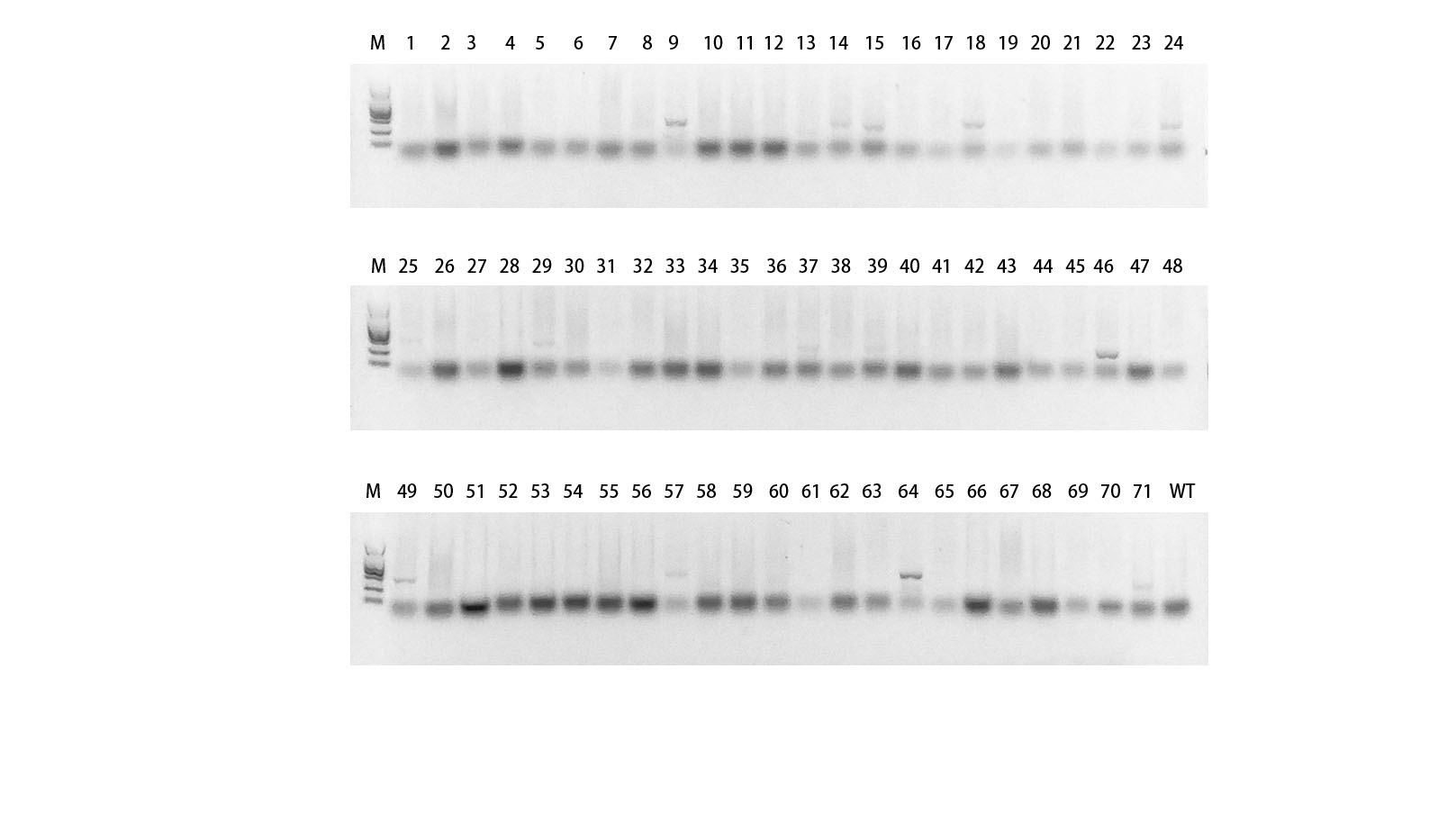
**

2000bp

500bp

250bp

100bp

2000bp

500bp

250bp

100bp

2000bp

500bp

250bp

100bp

***SlyRIN-MC* (deletion)**

**F**

**
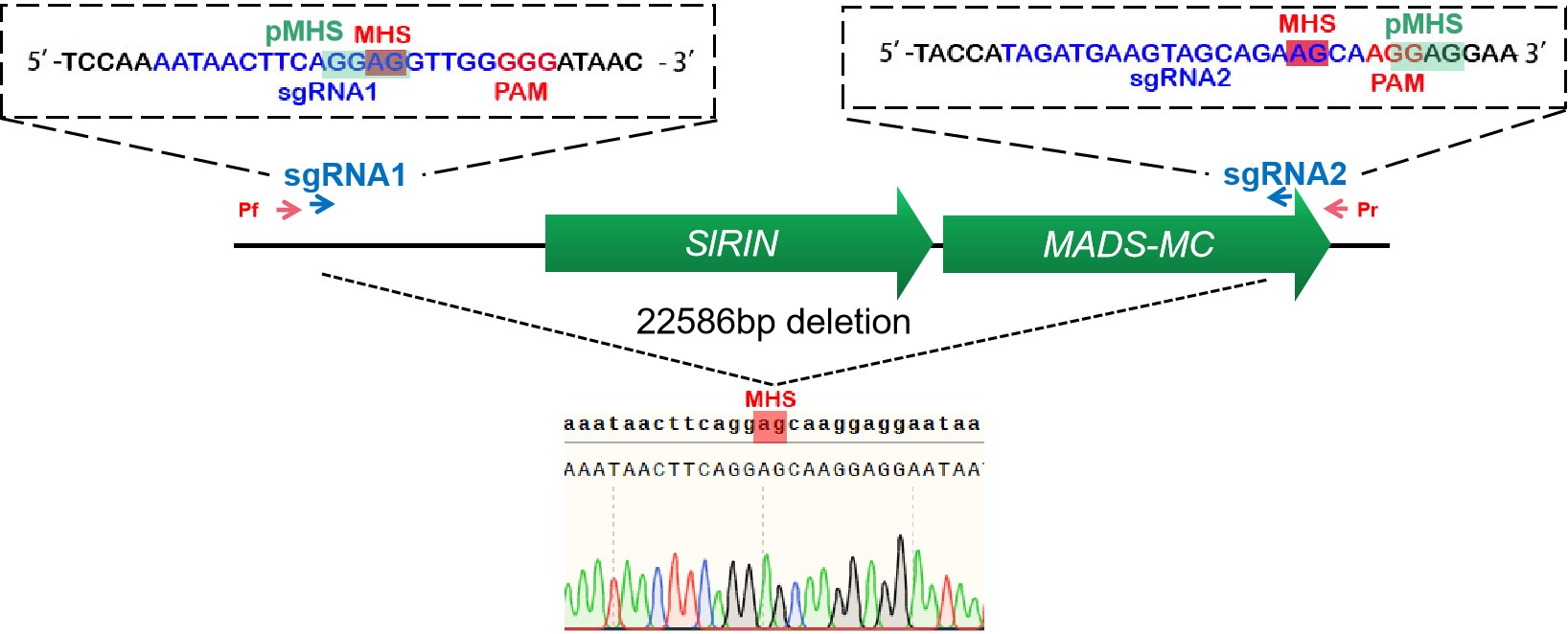
**

**Figure S1.** Identification of large fragment deletions in the MMEJ strategy by PCR

1. Efficiency of large fragment deletion at different genomic sites in the MMEJ .
2. Identification of large deletion of slyPDS by PCR (partial result) . The black triangle indicates the PCR product without large fragment deletion and the PCR product with large fragment deletion, respectively.
3. Identification of large deletion on sly ch.03 by PCR (partial result) . The black triangle indicates the PCR product with large fragment deletion.
4. Large fragment deletion of SlyCh.03 in the MMEJ strategy. The Pf/Pr primer pair detected large fragment deletions, and the predicted MHS are labeled as pMHS, while the actual repair sites are labeled in red.
5. Identification of large deletion on slyRIN-MC by PCR (partial result) . The black triangle indicates the PCR product with large fragment deletion .
6. Large fragment deletion of *slyRIN-MC* in the MMEJ strategy. The Pf/Pr primer pair detected large fragment deletions, and the predicted MHS are labeled as pMHS, while the actual repair sites are labeled in red.
