# Supplementary material for: Efficient large fragment deletion in plants: double pairs of sgRNAs are better than dual sgRNAs
Source: Hortic Res. 2023 Aug 22;10(10):uhad168. doi: 10.1093/hr/uhad168 (PMC10569238; doi:10.1093/hr/uhad168)
Supplement: Web_Material_uhad168 [file web_material_uhad168.zip › Figure S2.docx]

**Figure S2**

**A
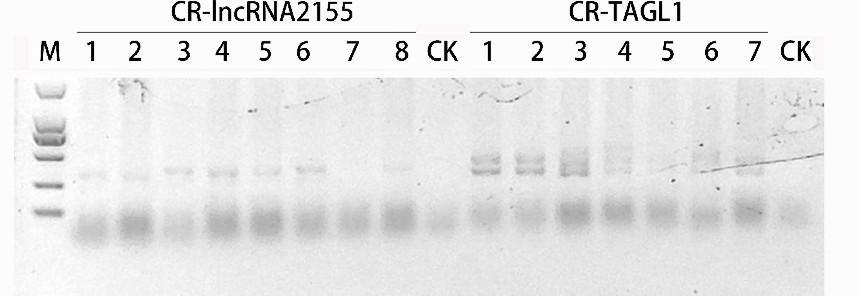
**

2000bp

500bp

250bp

100bp

***slyTAGL1* (Deletion)**

**lncRNA 2155 (Deletion)**

**B**


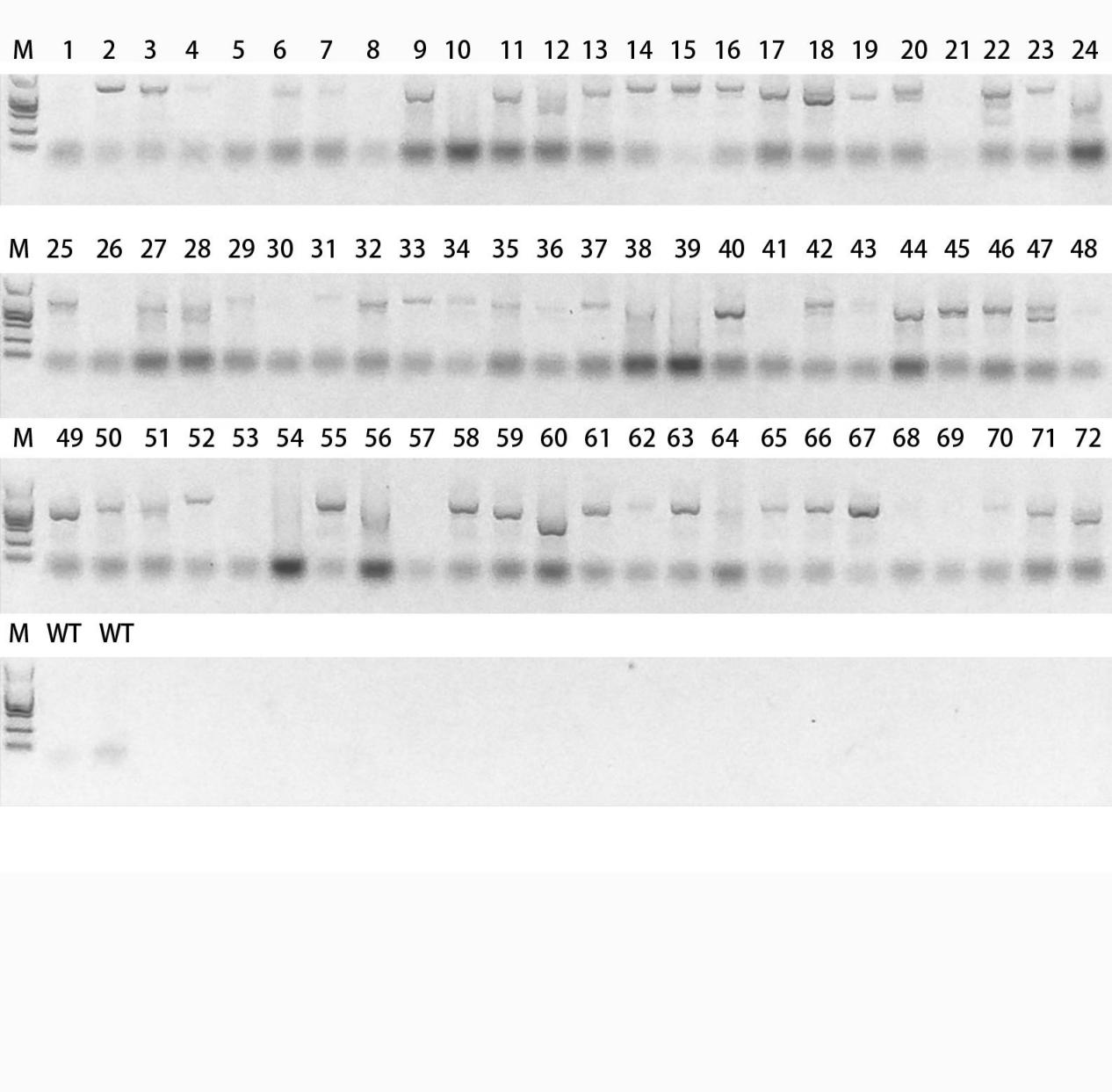


**Ch.03 (Deletion)**

2000bp

1000bp

250bp

100bp

2000bp

1000bp

250bp

100bp

2000bp

1000bp

250bp

100bp

2000bp

1000bp

250bp

100bp

**C**

**
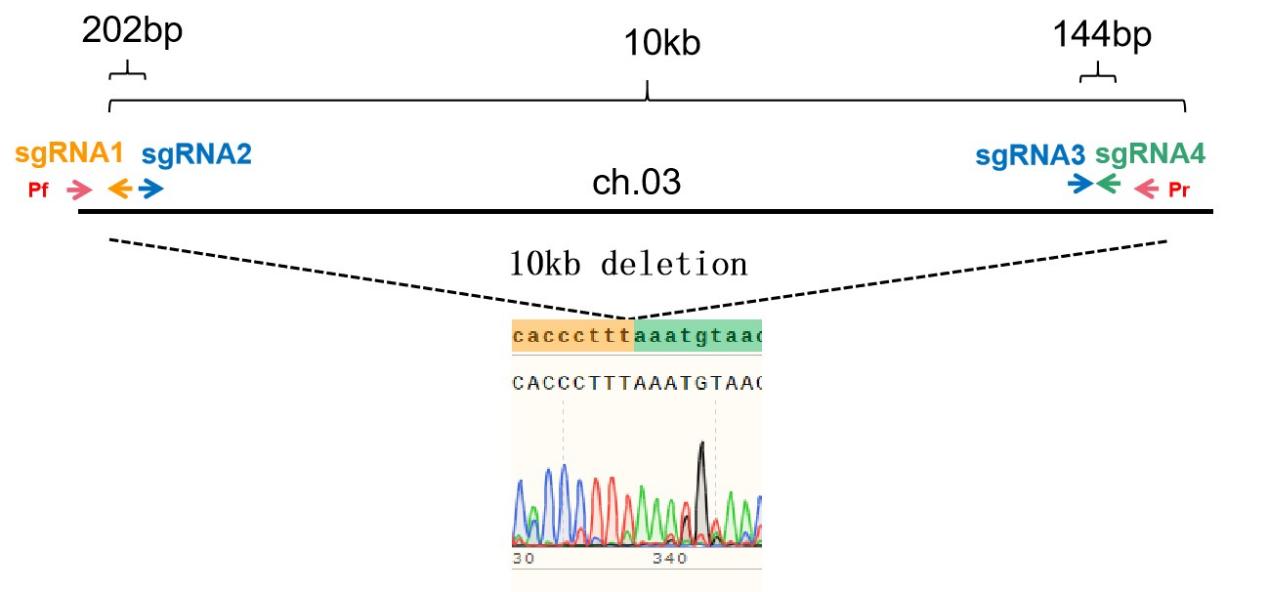
**

**Figure S2** Identification of large fragment deletions in the DPS strategy by PCR

1. Large deletions of lncRNA 2155 and *slyTAGL1* by transient infection in tomato cotyledonwere identified by PCR. The black triangle indicates the PCR product withr large fragment deletion.
2. Large deletions on slyCh.03 by stable transfection in tomato explants identified by PCR. The black triangle indicates the PCR product withr large fragment deletion.
3. Large fragment deletion of Ch.03 using DPS in transient transformation of tomato bud parts differentiated from the explants.
