# Supplementary material for: Efficient large fragment deletion in plants: double pairs of sgRNAs are better than dual sgRNAs
Source: Hortic Res. 2023 Aug 22;10(10):uhad168. doi: 10.1093/hr/uhad168 (PMC10569238; doi:10.1093/hr/uhad168)
Supplement: Web_Material_uhad168 [file web_material_uhad168.zip › Figure S3.docx]

**A**

**B**

**CR-9k**

2000bp

1000bp

750bp

100bp

CR-10k


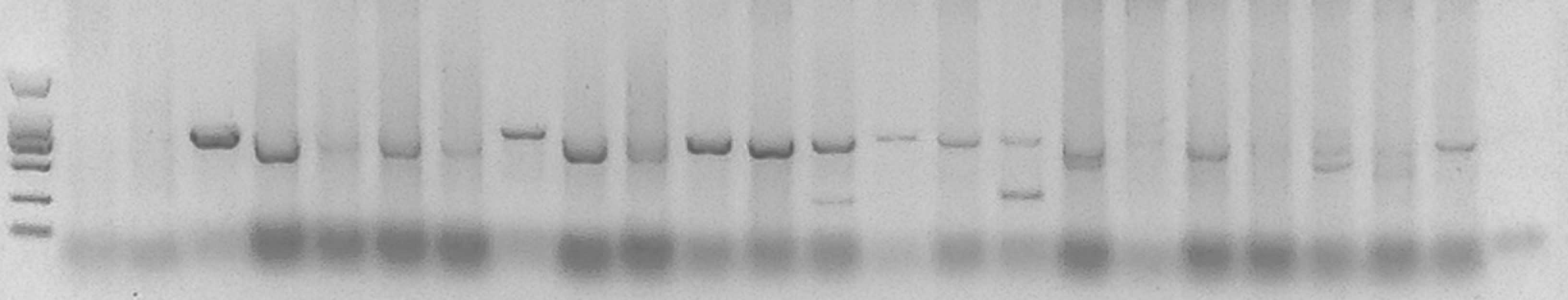


WT

**
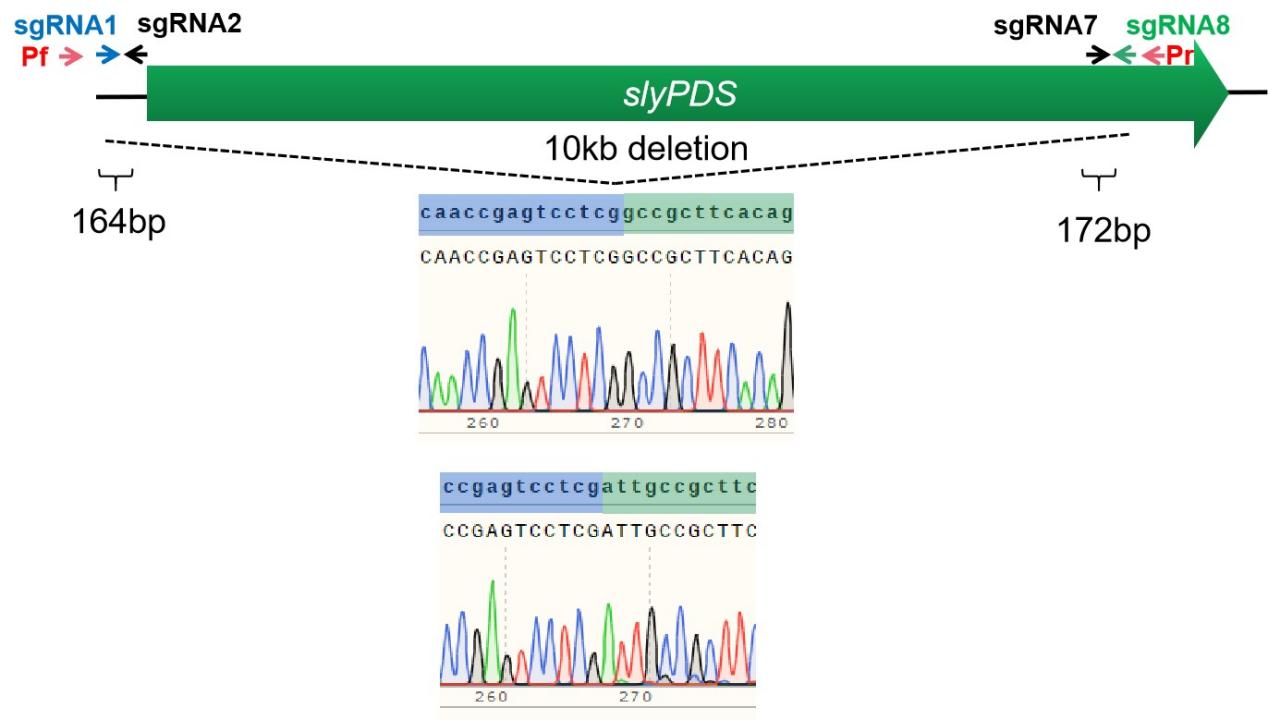
****C**

9kb deletion


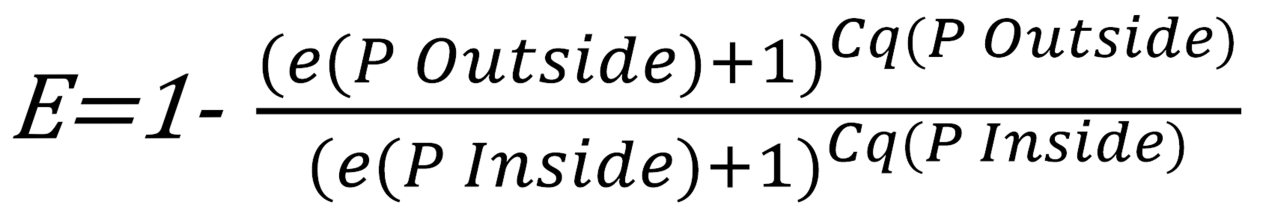


**D**

**
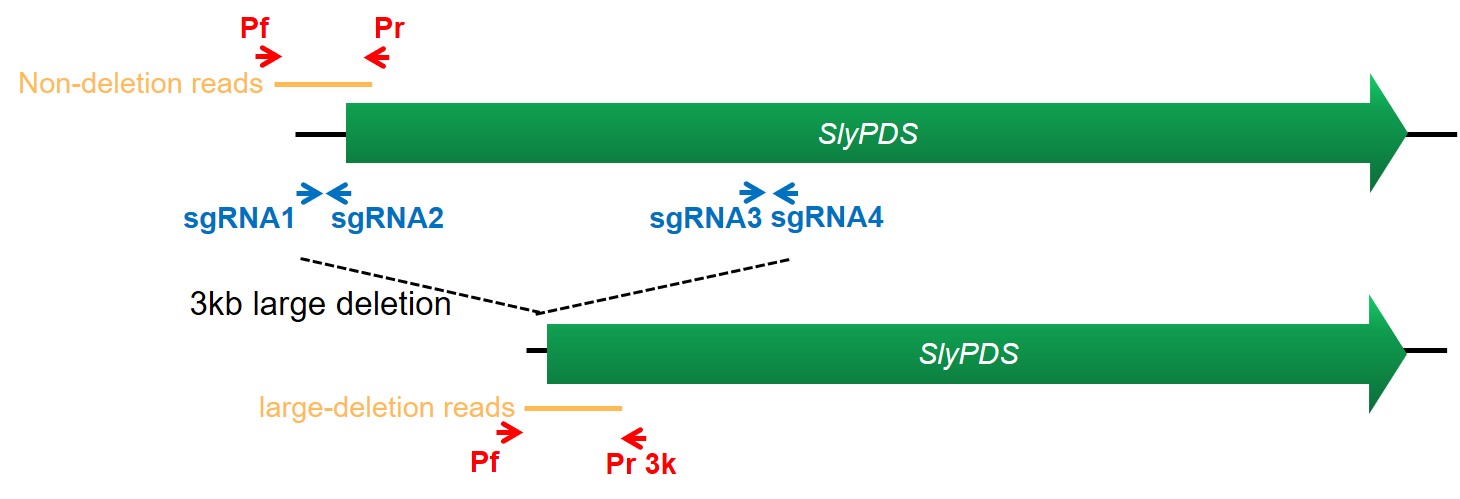
**

**E
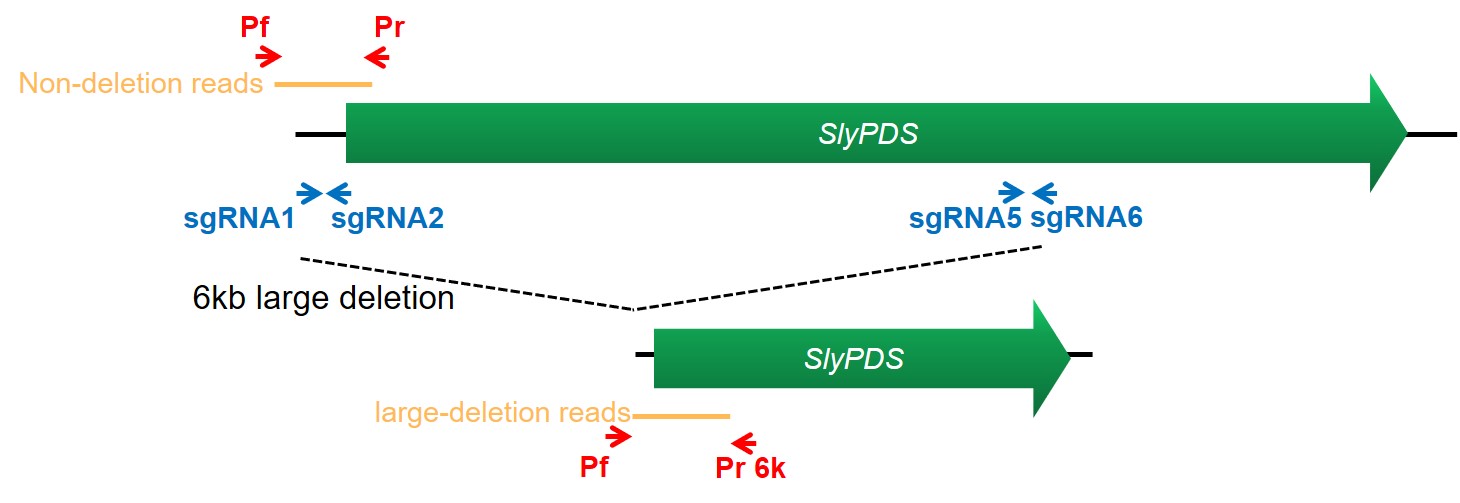
**

**F
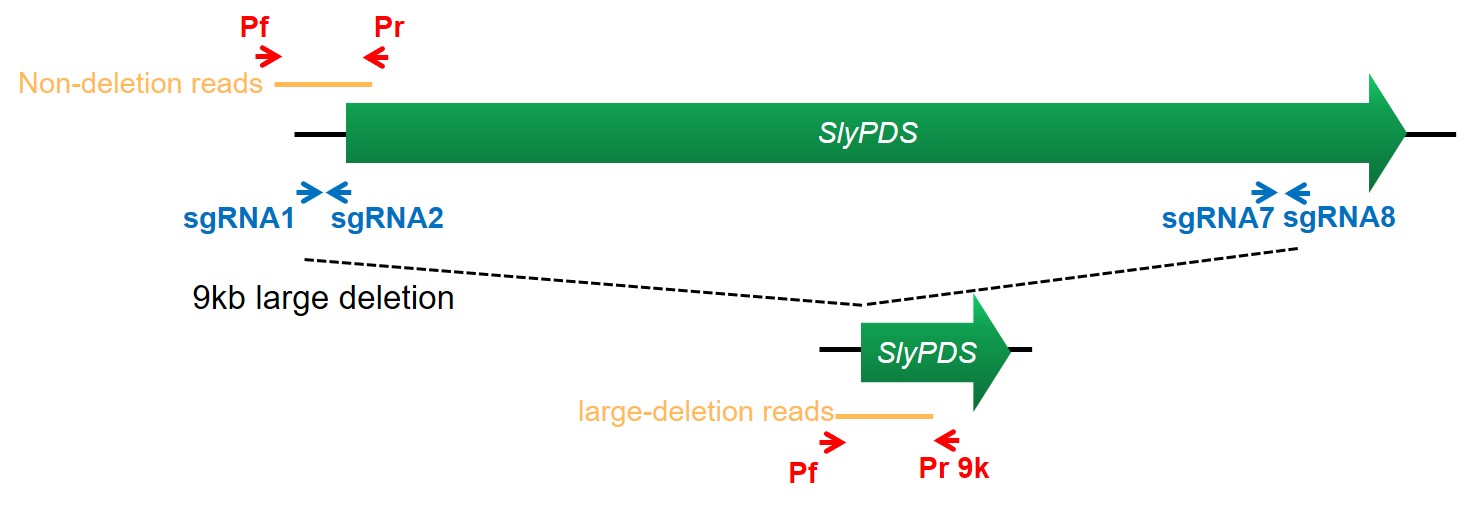
**

**G**


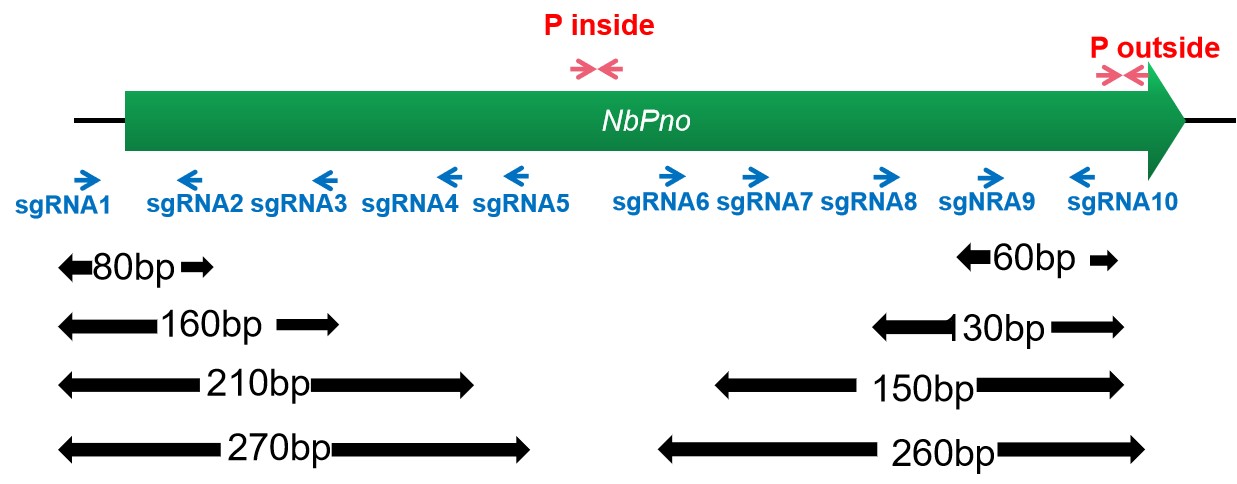


**H**

**Figure S3** Chromosome deletions occurred in DPS.

1. PCR was employed to detect large fragment deletions with 10kb spacing in PDS. Black triangles indicate partial sequencing samples.
2. Sequencing results of PCR products. All deletion events occurred between sgRNA1 and sgRNA8.
3. Formula for calculating the efficiency of large fragment deletion. E represents the efficiency of large fragment deletion; e is the efficiency of primer amplification; Cq is the cycle threshold.
4. Schematic diagram of next-generation sequencing of CR-3k.
5. Schematic diagram of next-generation sequencing of CR-6k.
6. Schematic diagram of next-generation sequencing of CR-9k and CR-2T.
7. Target sites on *NbPno* were used to analyze the effect of the internal spacing of single target site pairs on the efficiency of large fragment deletion. Five target site groups were established: NbPno-1 (sgRNA1, sgRNA2, sgRNA9, sgRNA10), NbPno-2 (sgRNA1, sgRNA3, sgRNA8, sgRNA10), NbPno-3 (sgRNA1, sgRNA4, sgRNA7, sgRNA10), NbPno-4 (sgRNA1, sgRNA5, sgRNA6, sgRNA10) and NbPno-2T (sgRNA1, sgRNA10).
8. In tobacco, qPCR analysis showed that the efficiency of large fragment deletion decreased with the increase in fragment length in a single target site pair. The bars represent mean values ± SD.
